# Expression of miR-29c, miR-93, and miR-429 as Potential Biomarkers for Detection of Early Stage Non-Small Lung Cancer

**DOI:** 10.1371/journal.pone.0087780

**Published:** 2014-02-11

**Authors:** Wangyu Zhu, Jianying He, Dongdong Chen, Bingjie Zhang, Liyun Xu, Haijie Ma, XiaoGuang Liu, YongKui Zhang, Hanbo Le

**Affiliations:** 1 Department of Joint Immunogenomics Laboratory, Zhoushan Hospital, Zhoushan, Zhejiang, China; 2 Department of Cardio-Thoracic Surgery, Zhoushan Hospital, Zhoushan, Zhejiang, China; Penn State University, United States of America

## Abstract

**Background:**

Altered expression of miRNA expression contributes to human carcinogenesis. This study was designed to detect aberrant miRNA expressions as a potential biomarker for early detection and prognosis prediction of non-small cell lung cancer (NSCLC).

**Methods:**

miRNA array was used to profile differentially expressed miRNAs and Taqman-based quantitative RT-PCR assays were used to analyze levels of miR-29c, miR-93, and miR-429 expression in NSCLC tissue samples, corresponding normal tissue samples, and serum samples from 70 NSCLC patients as well as in serum samples from 48 healthy controls.

**Results:**

Levels of miR-29c and miR-93 expression were upregulated in NSCLC tissues, while serum levels of miR-29c were also upregulated, but levels of serum miR-429 were decreased in NSCLC. Moreover, the levels of miR-429 expression in NSCLC tissues were associated with those in serum samples. Receiver operating characteristic (ROC) curve analysis showed that at the optimal cut-off point, the areas under the ROC curve for serum levels of miR-29c and miR-429 were 0.723 and 0.727, respectively, levels which are higher than that of carcinoma embryonic antigen (0.534) in diagnosis of stage I NSCLC. In addition, serum levels of miR-429 were associated with poor overall survival of NSCLC patients. Both univariate and multivariate analyses showed that serum miR-429 level was an independent prognostic predictor for NSCLC.

**Conclusions:**

The results of the current study suggest that detection of serum miR-29c and miR-429 expression should be further evaluated as a novel, non-invasive biomarker for early stage NSCLC.

## Introduction

MicroRNAs (miRNAs) are a class of small non-coding RNAs that can function as endogenous RNA interference to regulate expression of the targeted genes [1]–[3]. To date, a total of 1048 human miRNAs have been identified and reported in the RNA database, and this number continues to increase. Some of these miRNAs have been reported to be useful as potential biomarkers for diagnosis, prognosis, and personalized therapy of human cancers [1]–[6], because altered expression of these miRNAs contributes to human carcinogenesis. For example, aberrant miRNA expression has been reported in lung cancer. A number of researchers, including us, have reported the potential clinical application of circulating miRNAs (such as miR-1254, miR-142-3p, miR-24, miR-183, miR-21, miR-221, miR-29c, miR-486, miR-30d, miR-1, miR-499, and miR-210) [7], [8]. Thus, further investigation of aberrant miRNA expression could lead to the discovery of novel miRNA biomarkers for lung cancer.

Lung cancer is the leading cause of cancer-related deaths worldwide [9]. Despite advances in early detection and improvements in treatment options, patients with advanced disease still frequently develop recurrent disease after extended radical resections, and the 5-year overall survival rate is still only 5%. Thus, novel approaches to effectively managing this disease at the molecular level could identify patients who have a higher or lower risk of relapse following surgery and provide biomarkers for the prediction of patient survival. In this regard, the use of serum miRNAs as potential biomarkers for diagnosing and predicting prognosis of lung cancers has been reported [7], [10]. Indeed, several studies have identified miRNA signatures that differ between normal and cancerous tissues for the classification of cancer types and for tumor diagnosis and prognosis. It may also be possible to use miRNA expression as a biomarker to monitor treatment efficacy or predict cancer progression [11]–[12]. However, to date, most studies have focused on tumor tissue, and changes in circulating serum miRNAs levels and the relationship between these changes and the tissue at disease onset remain controversial [7]. Moreover, the usefulness of many circulating miRNAs has been demonstrated in the diagnosis of NSCLC, but few circulating miRNAs showed diagnostic value for early stage NSCLC [13], [14]. However, further verification in different populations is needed. Thus, in this study, we analyzed levels of three miRNAs (miR-29c, miR-93, and miR-429) in non-small cell lung cancer (NSCLC) tissues and compared them to those in serum samples of NSCLC patients and healthy controls, particularly, their expression levels in early stage NSCLC patients. Meanwhile, we analyzed the data by comparison with carcinoma embryonic antigen (CEA), which is a widely used marker in the diagnosis of NSCLC [15]. We then investigated associations between their expression and clinicopathological and survival data from NSCLC patients.

## Materials and Methods

### Ethics Statement

This study was approved by the Ethical Review Committee of Zhoushan Municipal Government of China, and all biological samples were obtained with patients' written informed consent.

### Patient samples

In this study, we recruited 70 patients with surgically resected NSCLC and matched distant noncancerous tissues from Zhoushan Hospital, Zhejiang, China between January 2008 and May 2009. There were 56 male and 14 female patients with NSCLC, including 34 patients younger than 60 years and 36 older than 60 years. Meanwhile, sera from all NSCLC patients and 48 healthy volunteers matched according to sex and age were also collected. None of the patients enrolled in this study had received any chemotherapy or radiotherapy before surgery. For tissue sample collection, upon removal of the surgical specimens, the tissues were immediately transported to the Pathology Laboratory, and the samples were placed in a cryovial, snap-frozen in liquid nitrogen for 30 min, and stored at −80°C until use. All tissue samples were reviewed by two pathologists, and the diagnosis was made according to the National Comprehensive Cancer Network (NCCN) criteria. There were 34 lung adenocarcinomas and 36 lung squamous cell carcinomas, and 46 tumors were moderately to highly differentiated, whereas 24 were poorly differentiated. Moreover, 18 NSCLC patients had a tumor less than 3 cm in size, whereas 52 patients with a tumor larger than 3 cm. Lymph node metastasis was found in 32 patients, and 36 patients had stage I NSCLC and 34 patients had stage II, III, or IV NSCLC (Table 1). In addition, serum levels of CEA were measured using chemiluminescence as part of routine clinical tests, and reference value was acquired from the clinical database at Zhoushan Hospital.

**Table 1:** Clinicopathological features of 70 NSCLC patients.

Clinical features	n
Mean age (years)	59
<60	34
≥60	36
Gender	
Male	56
Female	14
Tumor size	
0–3 cm	18
>3 cm	52
Histological classification	
Adenocarcinoma	34
SCC	36
Differentiation	
Moderate–well	46
Poor	24
Lymph node	
Negative	38
Positive	32
Stage classification	
Stage I	36
Stage II, III, and IV	34

### RNA isolation and quantitative RT-PCR

Total cellular RNA was isolated from 100 mg lung tissue or 600 µl serum samples using a miRNAs isolation kit or mirVana PARIS RNA isolation kit (Applied Biosystems, Foster City, CA, USA) according to the manufacturer's protocol. RNA concentration was determined using a NanoDrop ND-1000 spectrophotometer (NanoDrop Technologies, Wilmington, DE, USA), and RNA quality was measured using a denaturing 15% polyacrylamide gel. The reverse transcription reaction was carried out using the TaqMan MicroRNA Reverse Transcription Kit (Applied Biosystems) according to the manufacturer's instructions in a total reaction volume of 7.5 µl. qPCR was performed in triplicate using TaqMan 2× Universal PCR Master Mix without AmpErase UNG (Applied Biosystems) in an ABI 7500 Real-Time PCR system (Applied Biosystems) with the following conditions: 95°C for 10 min, followed by 40 cycles of 95°C for 15 s and 60°C for 1 min. The cycle threshold (Ct) values were calculated using SDS 2.0.1 software (Applied Biosystems). Template-free controls for both RT and PCR were included in each experiment to ensure target-specific amplification.

The average levels of miRNA expression in tissues and sera were normalized relative to the average amounts of U6 snRNA and U48 snRNA using the 2^−ΔΔCt^ method [16]–[17]. The mean Ct values for these five miRNAs were calculated excluding outliers (i.e., replicates with Ct values differing by more than one cycle from the median). In addition, if CtU6,ave and CtU48,ave each did not occur within 32 cycles, the assay was repeated. Samples with low U6 or U48 snRNA levels were not excluded from data analyses.

### Statistical analysis

All statistical analyses were performed using GraphPad Prism 5.0 software (GraphPad Software Inc., San Diego, CA, USA). The data were analyzed using homogeneity of variance. One-way ANOVA or Wilcoxon two-sample tests were used to test for associations between miRNA expression levels and clinicopathological features of the patients. A paired sample t-test was used to compare differences in miRNA expression between lung tissue and serum samples. Receiver operating characteristic (ROC) curves were generated to assess the diagnostic accuracy of each parameter. Patient survival was estimated by the Kaplan-Meier method, and the log-rank test was used to compare the survival between groups. The Cox hazard regression model was used to analyze the risk factors for NSCLC. All statistical tests were two-sided, and a *P* value of 0.05 was considered statistically significant.

## Results

### Differential expression of three miRNAs in NSCLC tissue and patient sera

**Figure 1:**
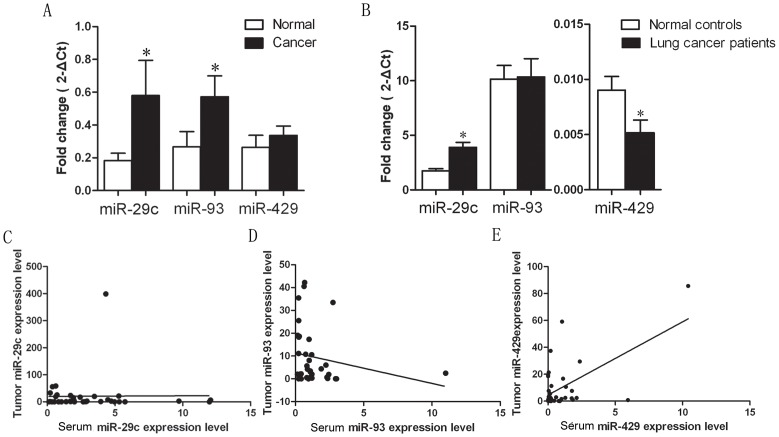
Differential expression of miRNAs in NSCLC. A, qRT-PCR detection of three miRNAs in 70 NSCLC tumors and the corresponding normal lung tissues. *P*-values for miR-29c, miR-93, and miR-429 were 0.0408, 0.0444, and 0.3903, respectively, using a paired sample *t*-test. B, qRT-PCR analysis of serum miRNA levels in serum samples of 70 NSCLC patients vs. 48 healthy controls. *P*-values of serum miR-29c, miR-93, and miR-429 were 0.0012, 0.9291, and 0.0001, respectively, using an unpaired sample t-test. C, D, E, Association of these miRNA levels between NSCLC tissue and serum samples. Pearson correlation test showed that miR-429 expression in serum was significantly associated with that in NSCLC tissues (r = 0.3578, *P* = 0.0024), whereas serum levels of miR-29c and miR-93 were not associated with those in NSCLC tissues (r = −0.07877, *P* = 0.5169 and r = 0.1515, *P* = 0.2105, respectively). *P<0.05 between groups.

Based on our miRNA array (Agilent) and validation data, we selected three miRNAs (miR-29c, miR-93, and miR-429) for further study in NSCLC samples. We performed qRT-PCR analysis for these three miRNAs in 70 pairs of NSCLC and corresponding noncancerous lung tissues. Our data showed that levels of miR-29c and miR-93 expression were upregulated in NSCLC tissues compared to the corresponding noncancerous lung tissues (*P* = 0.0408 and *P* = 0.0444, respectively), whereas miR-429 levels were not significantly different between NSCLC and noncancerous lung tissues (*P* = 0.3903, Figure 1A).

We then investigated whether these differences in miRNA expression were present in serum samples from these patients. We found that serum miR-93 expression did not differ between NSCLC patients and healthy controls (*P* = 0.3530). However, miR-29c expression was significantly increased serum from NSCLC patients (*P* = 0.0012), and miR-429 expression was significantly decreased (*P* = 0.0001, Figure 1B). The serum level of miR-429 expression was significantly correlated with that in NSCLC tissues (r = 0.3578, *P* = 0.0024, Figure 1E), whereas serum levels of miR-29c and miR-93 expression were not associated with those in NSCLC tissues (r = −0.07877, *P* = 0.5169 and r = 0.1515, *P* = 0.2105, respectively, Figure 1C and D).

### Associations between altered miRNA expression and clinical characteristics of NSCLC

**Figure 2:**
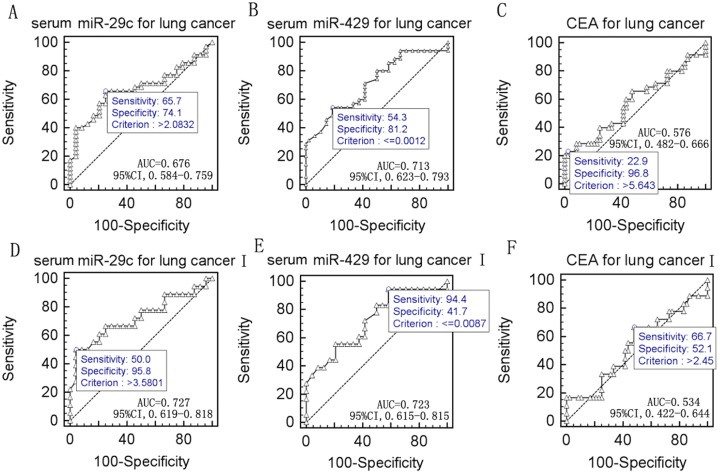
ROC curves for serum miRNA and CEA levels in NSCLC patients. A, miR-29c; B, miR-429; C, CEA showed ROC curves and an AUC with diagnostic power to distinguish NSCLC patients from healthy controls. D, miR-29c; E, miR-429; F, CEA showed ROC curves and an AUC with diagnostic power to distinguish stage I NSCLC patients from healthy controls.

We next investigated associations between the altered miRNA expression levels (miR-29c and miR-93 in NSCLC and miR-29c and miR-429 in serum) with the clinicopathological characteristics of the NSCLC patients. We found that increased miR-93 expression was strongly associated with NSCLC histology (*P* = 0.031, Table 2), whereas serum miR-29c expression was associated with abnormal CEA levels (*P* = 0.030, Table 2). In contrast, no other associations were detected between the expression levels of these miRNAs. Furthermore, we plotted expression data for miR-29c and miR-429 using ROC curves to identify a cut-off value that could distinguish lung cancer patients from healthy controls. ROC curve analysis showed that at the optimal cut-off, serum levels of miR-29c had a sensitivity of 65.7% and a specificity of 74.1% for distinguishing NSCLC patients from healthy controls with an area under the curve (AUC) of 0.676 (*P* = 0.0004, 95% confidence interval [CI]: 0.584–0.759, Figure 2A). In addition, serum levels of miR-429 had a sensitivity of 54.3% and a specificity of 81.2% for distinguishing NSCLC patients from healthy controls with an AUC of 0.713 (*P*<0.0001, 95% CI: 0.623–0.793, Figure 2B). Because the blood CEA test is a widely used marker for NSCLC, we compared CEA results with the miRNA expression levels and found that CEA had an AUC of 0.576 (*P* = 0.1493, 95% CI: 0.482–0.666, Figure 2C). However, for the combination of miR-29c expression and CEA, the data showed an AUC of 0.712 (*P*<0.0001, 95% CI: 0.622–0.792); for the combination of miR-429 expression and CEA, the AUC was 0.707 (*P* = 0.0007, 95% CI: 0.616–0.787); and for the combination of miR-29c expression and miR-429 with CEA, the AUC was 0.797 (*P*<0.0001, 95% CI: 0.713–0.865).

**Table 2:** Associations of aberrant miRNA expression with clinicopathological data of NSCLC patients.

		Tumor miR-29c			Tumor miR-93			Serum miR-29c			Serum miR-429		
	n	low	high	*P*	low	high	*P*	low	high	*P*	low	high	
Mean age (years)	59												
<60	34	18	16	0.811	18	16	0.811	15	19	0.473	15	19	0.473
≥60	36	17	19		17	19		20	16		20	16	
Gender													
Male	56	28	28	1.000	27	29	0.766	27	29	0.766	25	31	0.133
Female	14	7	7		8	6		8	6		10	4	
Tumor size													
0–3 cm	18	8	10	0.785	5	13	0.054	11	7	0.413	11	7	0.413
>3 cm	52	27	25		30	22		24	28		24	28	
Histological classification													
Adenocarcinoma	34	17	17	1.000	22	12	0.031*	14	20	0.232	16	18	0.811
SCC	36	18	18		13	23		21	15		19	17	
Differentiation													
Moderate-well	46	26	20	0.327	26	20	0.208	22	24	0.802	24	22	0.802
Poor	24	11	15		9	15		13	11		11	13	
Lymph node													
Negative	38	16	22	0.230	15	23	0.092	17	21	0.472	19	19	1.000
Positive	32	19	13		20	12		18	14		16	16	
Stage classification													
Stage I	36	20	16	0.473	16	20	0.473	16	20	0.473	20	16	0.473
Stage II, III, and IV	34	15	19		19	15		19	15		15	19	
CEA													
Normal	51	25	26	1.000	24	27	0.592	30	21	0.030*	27	24	0.592
Abnormal	19	10	9		11	8		5	14		8	11	

* *P*<0.05 between groups using χ^2^ test or Fisher exact test.

Moreover, we also found that serum levels of miR-29c had a sensitivity of 50.0% and a specificity of 95.8% for distinguishing stage I NSCLC from healthy controls with an AUC of 0.727 (*P* = 0.0001, 95% CI 0.619–0.818, Figure 2D). miR-429 had a sensitivity of 94.4% and a specificity of 41.7% for distinguishing stage I NSCLC patients from healthy controls with an AUC of 0.723 (*P* = 0.0001,95% CI 0.615–0.815, Figure 2E). CEA had a sensitivity of 66.7% and a specificity of 52.1% for distinguishing stage I NSCLC patients from healthy controls with an AUC of 0.534 (*P* = 0.6040, 95% CI 0.422–0.644, Figure 2F). In addition, the combination of miR-29c expression and CEA had an AUC of 0.757 (*P*<0.0001, 95% CI: 0.651–0.844), the combination of miR-429 expression and CEA had an AUC of 0.659 (*P* = 0.0128, 95% CI: 0.547–0.759), and the combination of miR-29c expression, miR-429 expression, and CEA had an AUC of 0.833 (*P*<0.0001, 95% CI: 0.736–0.906).

### Association between serum miR-429 level and survival of NSCLC patients

**Figure 3:**
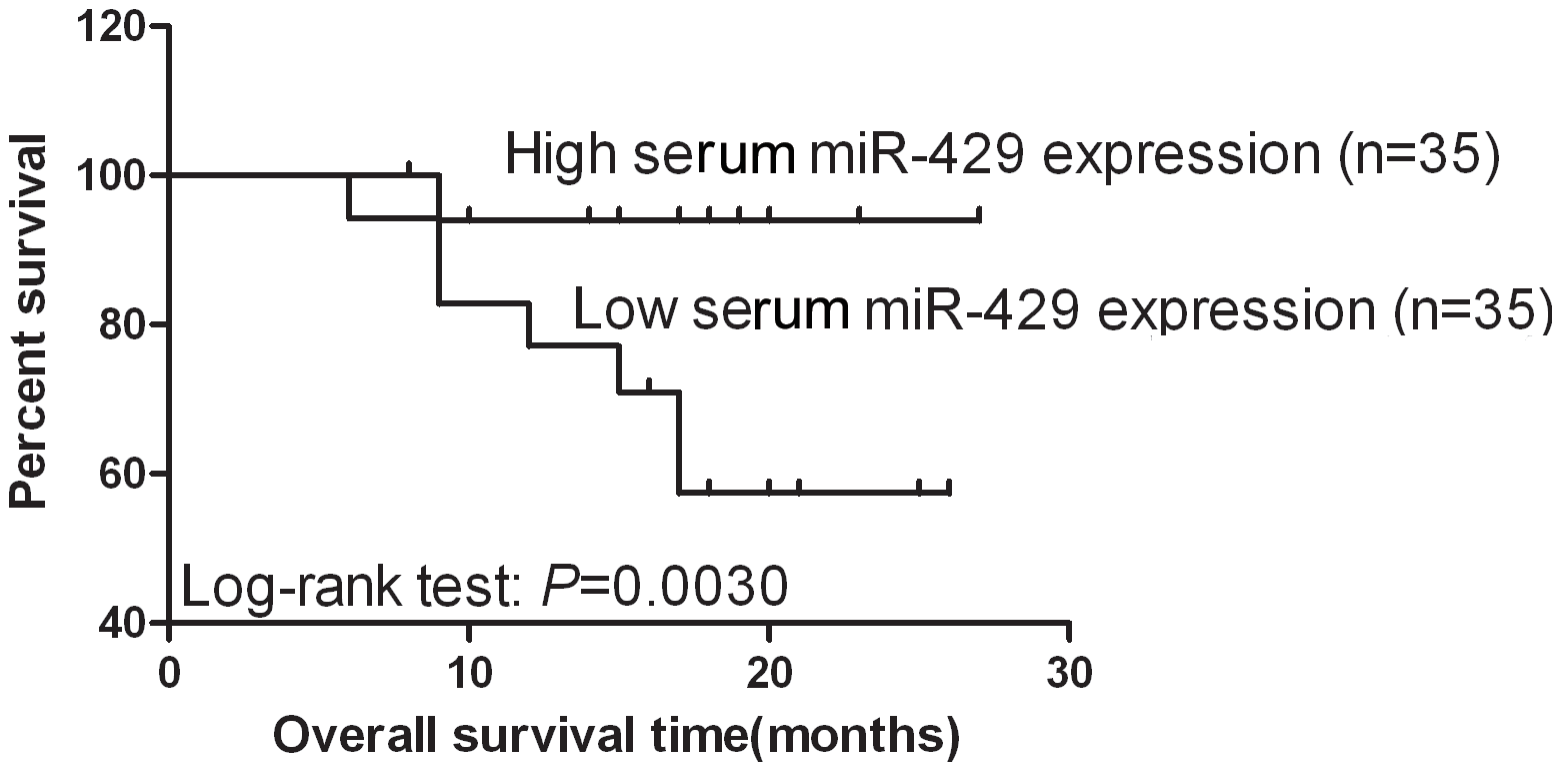
Kaplan-Meier survival curves for NSCLC patients according to serum level of miR-429. *P*-value for survival of patients with high and low levels of miRNA expression was calculated using the log-rank test. *P<0.05 between groups.

Because the expression of the three tested miRNAs was altered in NSCLC tissue and serum samples, we further evaluated whether detection of their levels could predict prognosis in NSCLC patients. We found that the serum level of miR-429 expression was associated with the overall survival of NSCLC patients (*P* = 0.0030 using a Log-rank test, Figure 3). However, expression of miR-29c, miR-93, and miR-429 in NSCLC tissues and serum levels of miR-29c and miR-93 were not associated with the overall survival of NSCLC patients. The univariate Cox hazard regression analysis demonstrated that serum levels of miR-429 were a significant prognostic indicator of NSCLC (adjusted hazard ratio = 6.458, 95% CI: 1.409–29.593, *P* = 0.016). The multivariate Cox proportional hazard regression analysis showed that serum levels of miR-429 were an independent prognostic predictor for NSCLC patients (hazard ratio = 12.875, 95% CI: 2.295–72.230, *P* = 0.004, Table 3).

**Table 3:** Univariate and multivariate Cox hazard regression analysis.

		Univariate analysis		Multivariate analysis	
	Levels	Adjusted hazard ratio (95% confidence interval)	*P*	Hazard ratio (95% confidence interval)	*P*
Tumor miR-29c	high/low	1.549 (0.518∼4.628)	0.433	0.974 (0.894∼1.061)	0.548
Tumor miR-93	high/low	0.672 (0.225∼2.010)	0.478	0.556 (0.152∼2.043)	0.377
Tumor miR-429	low/high	1.686 (0.570∼4.984)	0.345	2.749 (0.706∼10.707)	0.145
Serum miR-29c	high/low	0.563 (0.200∼1.579)	0.275	1.196 (0.342∼4.175)	0.779
Serum miR-93	high/low	0.656 (0.240∼1.796)	0.412	0.626 (0.230∼1.703)	0.359
Serum miR-429	low/high	6.458 (1.409∼29.593)	0.016*	12.875 (2.295∼72.230)	0.004*

* *P*<0.05.

## Discussion

In this study, we analyzed the expression of three miRNAs, selected based on our previous miRNA array and validation data, in NSCLC tissue and serum samples for association with clinicopathological and survival data from patients. We found that miR-29c and miR-93 expression was upregulated in NSCLC tissues compared to the corresponding noncancerous lung tissues. In addition, serum levels of miR-29c were significantly increased and serum levels of miR-429 were significantly decreased in stage I NSCLC compared to levels in 48 healthy controls. Furthermore, we found that lower serum miR-429 levels were associated with poor overall survival of NSCLC patients. The univariate and multivariate analyses showed that serum level of miR-429 was an independent predictor of the overall survival of NSCLC patients. Our current data indicate that detection of serum miR-29c and miR-429 expression should be further evaluated as a novel non-invasive biomarker for early stage of NSCLC.

Indeed, to date, a large number of studies have demonstrated that altered expression of specific miRNAs plays an important role in human tumorigenesis due to the functions of these miRNAs as gene suppressors or oncogenes [17]–[19]. Thus, detection of aberrant miRNA expression levels could be used for early diagnosis of human cancers or prediction of prognosis because miRNAs are more stable and resistant to RNase A digestion and other harsh conditions than are traditional protein markers [7], [10], [17], [20]. For example, Chen *et al* [10] showed that miRNAs are stable in circulating serum and can serve as potential diagnostic markers for lung cancer. Hu *et al* [7] reported that four miRNAs are independent predictors of overall survival in lung cancer patients. Le and colleagues [20] showed that serum levels of miRNAs are different in pre- and post-operative lung cancer samples and that these miRNAs can potentially be used as biomarkers for disease recurrence after surgery. However, only a few studies have focused on the diagnostic role of miRNAs in early stage NSCLC [13], [14]. In the current study, we analyzed the expression of three miRNAs in NSCLC tissues and serum samples for their potential value in the early diagnosis and prognosis of NSCLC patients. Our data showed that levels of miR-29c and miR-93 expression were upregulated in NSCLC tissues compared to the corresponding noncancerous lung tissues. Moreover, ROC data showed that the AUCs for miR-29c and miR-429 were 0.723 and 0.727, respectively, and these values were significantly higher than that for CEA (0.534), in stage I NSCLC patients, suggesting these miRNAs could be used for early diagnosis of NSCLC. CEA is a widely used biomarker in diagnosing NSCLC and was chosen as a positive control [15]. Indeed, Heegaard *et al* [8] showed that expression of miR-29c was significantly increased in 220 early stage NSCLC patients compared to 220 matched controls. In other studies, miR-93 expression was found to promote tumor angiogenesis and metastasis by suppressing large tumor suppressor homology 2 expression [21]–[22]. However, miR-429 is a member of the miR-200 family that was previously shown to inhibit the epithelial-to-mesenchymal transition by regulating E-cadherin, ZEB1, and SIP1 expression [23]–[25]. No study has yet revealed its role in the diagnosis and prognosis of NSCLC. In the present study, we for the first time showed that the expression of miR-429 in serum was downregulated in NSCLC patients compared to healthy controls and that lower miR-429 expression was associated with poor overall survival of NSCLC patients, suggesting miR-429 expression is an independent prognostic indicator for NSCLC. In addition, we found that serum levels of miR-429 were significantly associated with miR-429 expression in NSCLC tissues, which led to the hypothesis that tumor cells may utilize miRNAs to transport genetic information to the surrounding or distant cells and thereby promote tumor growth and progression [26]. In our previous study, we reported that serum and tumor levels of miR-96 were associated [18], supporting the notion that some miRNAs could be secreted from tumor tissue and promote cancer progression, which is also consistent with reports from Mitchell *et al* [17] and Hu *et al* [7].

Several studies have shown that detection of serum miRNAs could be particularly useful for the earlier diagnosis of clinically asymptomatic cancers [27]–[29]. For example, in previous lung cancer studies, levels of miR-1254, miR-574-5p, miR-486, miR-30d, miR-1, and miR-499 expression were significantly dysregulated in early-stage lung cancer [7], [30]. Chen *et al* [31] demonstrated through profiling that 10 serum miRNAs can serve as novel noninvasive biomarkers for lung cancer diagnosis. Keller *et al* [32] also suggested that developing lung cancer might be detectable years prior to diagnosis through a specific miRNA signature and that this signature changes during tumor development. Partly consistent with the results of these studies, the results of our current study demonstrate the usefulness of the three tested miRNAs, which were more sensitive than CEA for detecting stage I NSCLC. However, our current data are proof-of-principle, and further prospective study using a larger sample size will verify whether detection of these miRNAs is useful for early diagnosis of stage I NSCLC and survival prediction in NSCLC patients.

We found differences in the expression of three miRNAs between NSCLC tissues and corresponding noncancerous lung tissues. Importantly, we identified miR-29c and miR-429 as dysregulated in circulating serum samples from patients with early stage NSCLC, and the expression of these miRNAs was more sensitive than serum CEA levels in distinguishing NSCLC patients from healthy controls. Moreover, we also identified decreased serum miR-429 expression as an independent prognostic biomarker for NSCLC. The clinical value of the three tested miRNAs in the diagnosis and prognosis of NSCLC warrants further examination in larger cohorts.
